# Evaluation of Autografts Used in Anterior Cruciate Ligament Reconstruction in Terms of Tensile Strength

**DOI:** 10.7759/cureus.39927

**Published:** 2023-06-03

**Authors:** Ahmet Mert, Selim Cinaroglu, Hacı Keleş, Murat Aydin, Fatih Çiçek

**Affiliations:** 1 Department of Orthopedics and Traumatology, Niğde Ömer Halisdemir University Faculty of Medicine, Niğde, TUR; 2 Department of Anatomy, Niğde Ömer Halisdemir University Faculty of Medicine, Niğde, TUR

**Keywords:** quadriceps tendon, tensile strength, reconstruction, autograft, anterior cruciate ligament (acl)

## Abstract

Introduction

Anterior cruciate ligament (ACL) injuries increase the likelihood of chronic knee problems in later years, including early onset osteoarthritis. Therefore, ACL treatment is important in preventing knee problems from developing. The treatment of choice for ACL tears is surgery (ACL reconstruction), and the most popular tendons for ACL reconstruction are the patellar tendon, hamstring tendon (semitendinosus and gracilis tendons), and bone-patellar tendon-bone. The present study compares the tensile strength of autografts used in ACL reconstruction to identify the optimum autograft for ACL in terms of mechanical properties.

Methods

Cadavers were dissected, and the Achilles tendons, quadriceps tendons, hamstring tendons (semitendinosus and gracilis tendons), patellar tendon grafts, and ACLs were harvested. Tensile tests of each tendon graft were performed using a Shimadzu Autograph AG-IS 100 kN tester (Shimadzu, Kyoto, Japan).

Results

The mean difference in tensile strength between ACL and other grafts was lowest for the quadriceps in both males and females (p ˂ 0.001), meaning that ACL and quadriceps grafts are more compatible than other tendon grafts in terms of tensile strength.

Conclusion

The present study found the lowest mean difference in tensile strength to be between the ACL and the quadriceps tendon, suggesting that the use of the quadriceps tendon in ACL reconstruction will yield more positive outcomes.

## Introduction

Currently, anterior cruciate ligament (ACL) surgery is one of the most studied musculoskeletal system treatments in the field of orthopedics [1]. ACL injuries are common not only in people who engage in sports but also in the general population. The estimated incidence, while increasing every day, is 75/100,000 in the general population, with more than 200,000 ACL injuries recorded every year in the United States [2]. Around 60% of injuries sustained in sporting activities occur in the knee, and 50% of these are cruciate ligament injuries. ACL injuries are the most common injury sustained by professional athletes across all sports [3]. The ACL (ligamentum cruciatum anterior) is located in the anterolateral aspect of the ligamentum cruciatum posterius, with the lower end attached to the anterior intercondylar area of the tibia and the upper end to the posterior part of the inner surface of the condylus lateralis in a fan-like form [4]. The ACL is functionally and anatomically composed of the anteromedial and posterolateral bundles, although studies in the literature have reported a rate of 26% for a single ACL [5].

The ACL plays a crucial role in joint stability, and it is also the main structure that restricts the anterior movement of the tibia relative to the femur. The anterior movement of the tibia relative to the femur is four times greater in ACL-deficient knees than in normal knees [6].

While the ACL is the most commonly injured knee ligament, isolated ACL injuries are rare and damage the surrounding tissue. An ACL injury increases not only the risk of subsequent knee injuries, but also the likelihood of secondary injuries and chronic knee problems, including early onset osteoarthritis, in later years. The prevention and treatment of ACL injuries should thus be considered important [7]. The treatment of choice for ACL tears is surgery (ACL reconstruction) due to the failure of non-surgical procedures [2]. Allografts and autografts are frequently used in ACL reconstruction as the use of prosthetic ligaments can have adverse outcomes. The advantages of autografts include low risk of inflammatory reaction and almost no risk of disease transmission. Allografts, on the other hand, prevent donor site morbidity, shorten the operative time, and reduce postoperative pain [8]. It has been reported in the literature that autografts appear to be superior to allografts for ACL reconstruction in active patients [9], and the most commonly preferred tendons for ACL reconstruction are the patellar and hamstring (semitendinosus and gracilis) tendons and the bone-patellar tendon-bone (BPTB) [8], although the Achilles, quadriceps, tibialis anterior, tibialis posterior, and peroneus longus tendons can also be used as grafts in ACL reconstruction [10]. In order to fully identify the biomechanical behavior of a normal ACL, or a substitute soft tissue graft for that matter, both its structural and mechanical properties must be known. Mechanical properties characterize the behavior of the isolated graft material and can also be determined by a tensile loading test [11]. Tensile tests determine the mechanical behaviors of materials and are very important in engineering. The test continues until the rupture of the tested sample or until the maximum tension of the sample has been calculated [12].

The present study compares the autografts used in ACL reconstruction and ACL based on tensile strength and establishes the optimum autograft for ACL in terms of mechanical properties.

## Materials and methods

**Figure 1:**
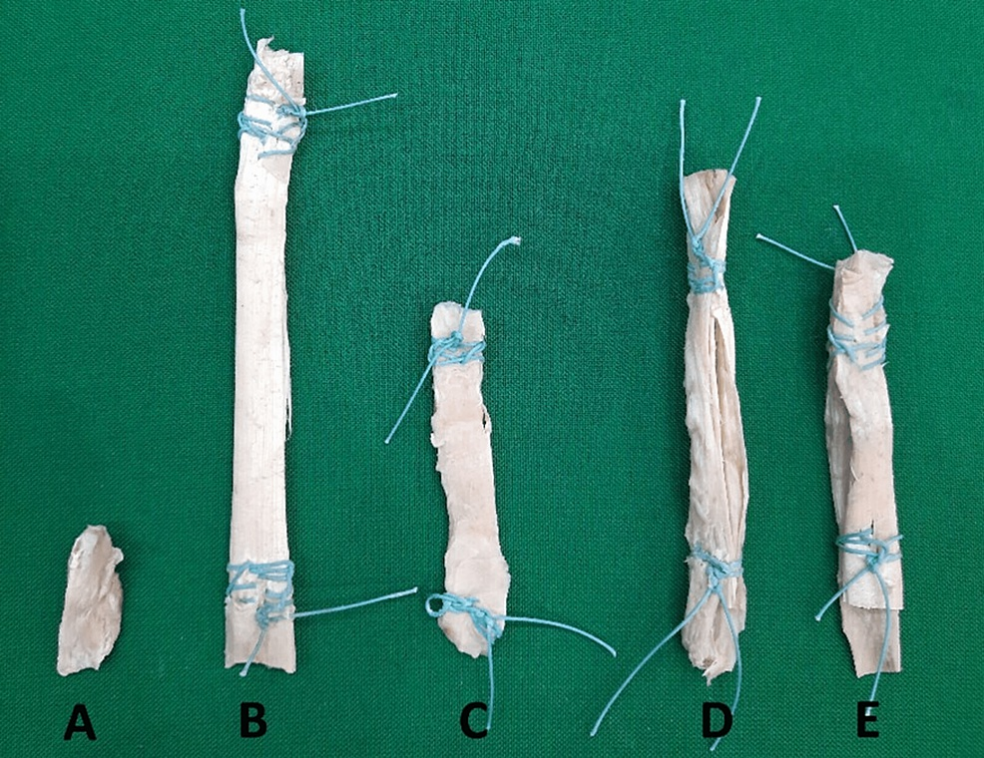
Tendon grafts harvested from cadavers (A) ACL. (B) Achilles tendon graft. (C) Patellar tendon graft. (D) Hamstring tendon graft. (E) Quadriceps tendon graft. ACL, anterior cruciate ligament

Ethics committee approval of this study was obtained with the approval numbered 2021/95 of Niğde Ömer Halisdemir University Ethics Committee. A total of 12 cadavers, six male and six female, over the age of 68 years (mean age: 72 years) were used in the study and were dissected to harvest the Achilles tendons, quadriceps tendons, hamstring tendons (semitendinosus and gracilis tendons), and patellar tendon grafts used in ACL construction, as well as the ACLs (Figure 1). The ACLs were harvested not in the form of a bone-tendon-bone (BTB) but by cutting both ends from the attachment line to the bones. To standardize the formaldehyde absorption rates of the harvested grafts, each tendon graft was kept in a 10% formaldehyde solution [13] for two weeks.

**Figure 2:**
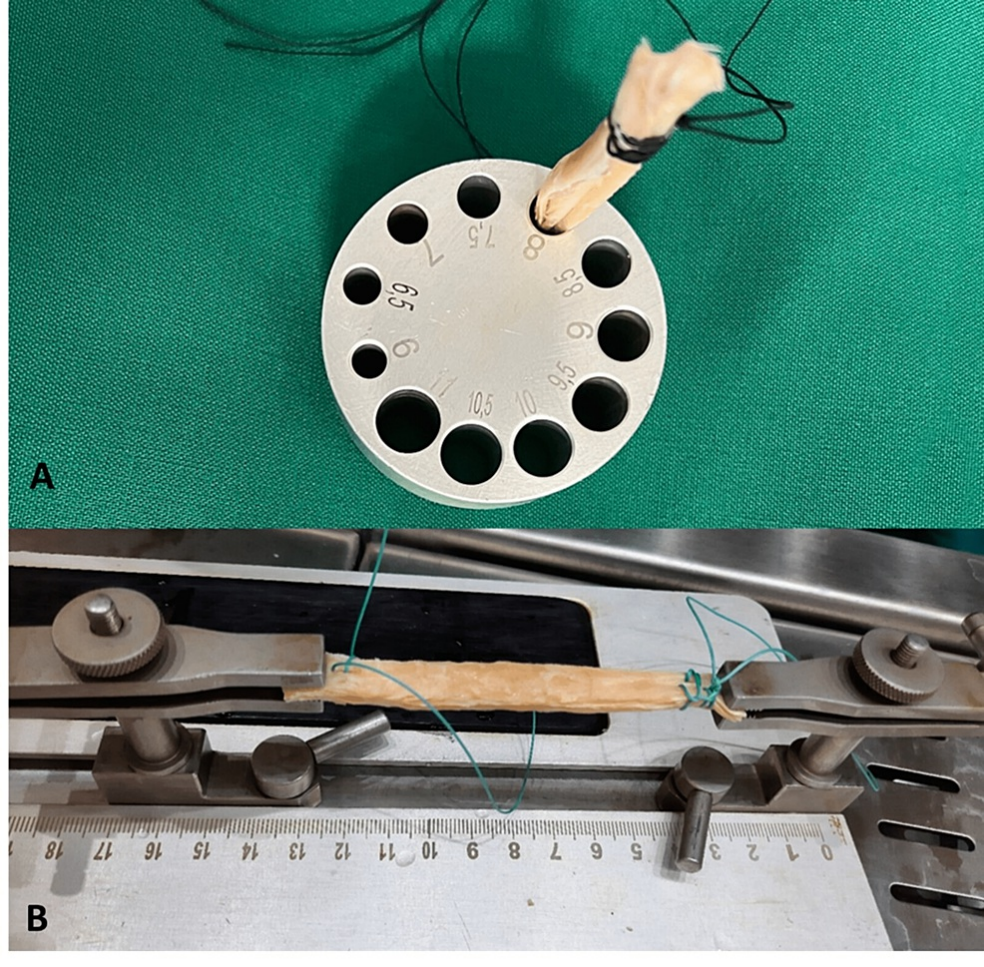
Preparing 8-mm grafts (A) Using a gauge. (B) The grafts are sutured from both ends with Ethibond sutures and made ready for ACL reconstruction. ACL, anterior cruciate ligament

The obtained grafts were appropriately prepared as 8-mm samples by orthopedic surgeons using a gauge used in ACL reconstruction and were made ready for ACL reconstruction by suturing them at both ends with Ethibond sutures (Johnson & Johnson, New Brunswick, NJ) (Figure 2).

**Figure 3:**
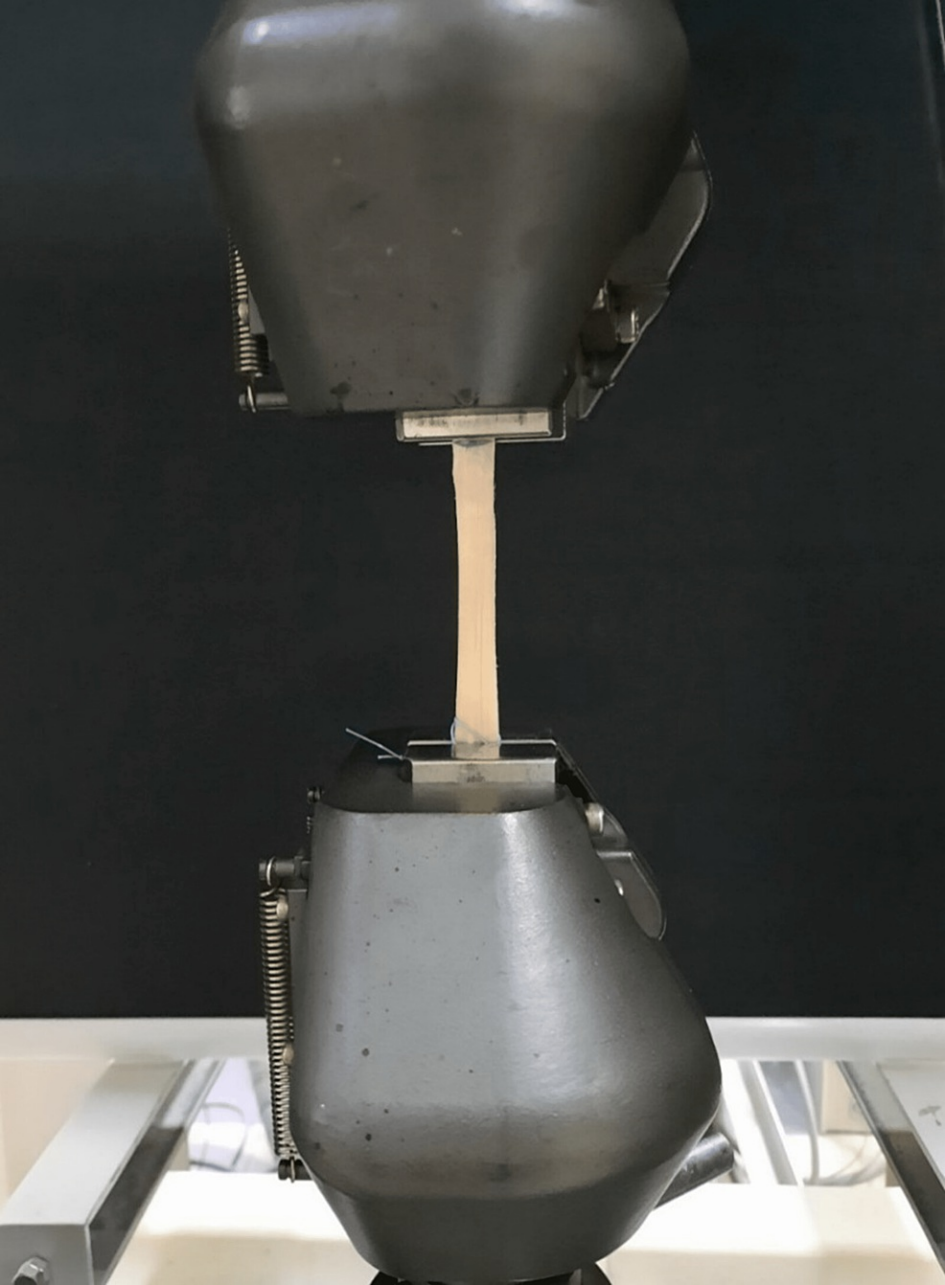
Vertical placement of the graft on the poles of the device

The tensile test was performed using a Shimadzu Autograph AG-IS 100 kN testing instrument (Shimadzu, Kyoto, Japan), and the procedure was carried out at a rate of 2 mm per minute. The grafts were placed vertically to the poles of the device (Figure 3), and a tensile load was applied at 2 mm per minute, and the peak tensile strength value was recorded individually for each graft.

The obtained data were assessed statistically using the analysis of variance and post-hoc tests using IBM SPSS Statistics for Windows (Trial Copy, Version 23.0, IBM Corp., Armonk, NY).

## Results

The results of the tensile tests of the ACL and muscular tendon samples for use as substitutes for ACL are presented in Table 1.

**Table 1:** Analysis of the results of the tensile strength test of grafts ACL, anterior cruciate ligament

		N	Mean	Std. Error	Min.	Max.	95% Confidence Interval	
							Lower Bound	Upper Bound
Load (N)	Achilles (male)	12	868.52	53.09	706.25	1262.5	751.65	985.38
	Achilles (female)	12	786.26	31.27	684.6	1007.3	717.43	855.09
	ACL (male)	12	70.83	1.64	63.25	79.81	67.21	74.45
	ACL (female)	12	69.70	1.7	58.7	81.7	65.94	73.45
	Hamstring (male)	12	807.07	40.82	662.50	1128.12	717.21	896.93
	Hamstring (female)	12	820.45	38.54	647.35	1032.1	735.61	905.28
	Patella (male)	12	405.18	15.92	312.50	525.0	370.13	440.22
	Patella (female)	12	312.96	18.97	233.12	434.37	271.21	354.72
	Quadriceps (male)	12	315.13	13.50	220.81	391.87	285.41	344.86
	Quadriceps (female)	12	278.38	17.98	187.5	401.67	238.81	317.96

Table 1 shows the tensile strength values for the Achilles, ACL, hamstring, patellar, and quadriceps grafts in males and females. As shown in Table 1, the Achilles tendon was the most resistant in terms of tensile strength to the applied loads or forces in males, while the ACL was the most sensitive. In females, the hamstring tendon was the most resistant in terms of tensile strength, and the most sensitive sample was the ACL (Table 1). A comparison of tensile strength between the ACL and other grafts is presented in Table 2.

**Table 2:** Comparison of tensile strengths of ACL and other grafts ACL, anterior cruciate ligament

Dependent Variable		Tendons	Mean Difference	Std. Error	95% Confidence Interval		p-Value
					Lower Bound	Upper Bound	
Load (N)	ACL (male)	Achilles	797.68	44.38	708.74	886.63	<0.01
		Hamstring	736.23	44.38	647.29	825.18	<0.01
		Patella	334.34	44.38	245.39	423.29	<0.01
		Quadriceps	244.3	44.38	155.35	333.24	<0.01
	ACL (female)	Achilles	716.55	35.49	645.42	787.69	<0.01
		Hamstring	750.75	35.49	679.61	821.88	<0.01
		Patella	243.26	35.49	172.12	314.39	<0.01
		Quadriceps	208.68	35.49	137.55	279.82	<0.01

As can be seen in Table 2, there was a significant difference in the tensile strengths of the ACL and other grafts in both genders (p ˂ 0.01). In both males and females, the mean difference in tensile strength between the ACL and other grafts was the lowest for the quadriceps sample (p ˂ 0.01). The mean difference in tensile strength between the ACL and other grafts was the lowest for the Achilles tendon in males and the hamstring in females, which and was statistically significant (p ˂ 0.01). Accordingly, quadriceps grafts can be considered more compatible with ACL than other tendon grafts in terms of tensile strength.

## Discussion

The present study has compared the tensile strength of the ACL with that of Achilles, hamstring, patellar, and quadriceps tendon grafts as potential substitutes for ACL in terms of tensile strength. All grafts used as substitutes for ACL were prepared as 8-mm samples. Considering the response of the grafts to tensile forces, it was observed that the ACL was the graft with the lowest resistance to tensile force in both genders, while the structure with the highest resistance (most resistant) was the Achilles graft in men and the hamstring graft in women. When the tensile strengths of the ACL and the other grafts were compared, a significant difference was noted among all (Table 2, p ˂ 0.01). The graft with the lowest mean difference from the ACL was the quadriceps graft in both genders, while the sample with the highest mean difference was the Achilles graft in males and hamstring in females.

The ACL has been reported to be the most commonly damaged knee ligament and increases the risk of subsequent knee injuries while also increasing chronic knee problems including early onset osteoarthritis, later in life. The treatment of ACL can thus be considered important [7]. The treatment of choice for ACL tears is surgery (ACL reconstruction) due to the lack of success associated with non-surgical procedures [2]. It has been reported that both structural and mechanical properties can be determined through tensile loading tests when selecting autografts for use in ACL reconstruction [11]. Lin et al. identified many studies reporting on the selection of grafts to be used in ACL reconstruction and underlined the lack of an ideal graft [14]. The authors also reported on the absence of a generally accepted algorithm for graft selection for particular patient groups. In general, it has been expressed that the autograft of choice should be BPTB autograft and quadriceps autograft for the youngest and most active patients, while hamstring autografts should often be preferred for moderate-category patients who are moderately active but not necessarily elite athletes and who are still physiologically young. Finally, hamstring autografts are recommended for older, less active patients if non-surgical treatments fail [11,14]. Woo et al. reported on the reduction of the structural properties of the ACL with age and a mean tensile strength of 2,160 N for the ACL, 1,784 N for patellar tendon grafts, and 2,422 N for hamstring tendon grafts in a sample aged 22-35 years [15]. Trent et al. conducted a study using fresh frozen cadavers aged 25-55 years and found the maximum tensile strength of the ACL to be 633 N, while the study by Noyes and Grood based on autopsy samples reported the tensile strength of ACL to be 1730±660 N in the 16- to 26-year age group and 734±266 N in the 48- to 86-year age group [16,17]. Our study used samples from cadavers with a mean age of 72 years that were fixed in 10% formaldehyde solution, and we recorded a mean tensile strength of 70.83 N for the ACL, 405.18 N for the patellar tendon graft, and 807.07 N for the hamstring tendon graft in males, and 69.70 N for ACL, 312.96 N for the patellar tendon graft, and 820.45 N for the hamstring tendon in females. The lower tensile strength values obtained in the present study than those reported in other studies may be attributable to the age of the cadavers, the storage of the grafts in a 10% formaldehyde solution, the preparation of all grafts at a level (8 mm wide) that can be used in ACL reconstruction, and the quality of the instruments, especially for the measurement of tensile strength.

West and Harner commented on the difficulty in comparing the biomechanical properties of ACL grafts, which may vary significantly depending on the age of the donor, the size of the graft, and the testing methods [18]. The authors also commented on the quality of the clamp used during testing, which may allow the graft to slip during testing and may even crush the graft, resulting in premature failure as well as potential errors in the elongation measurements of the graft and lower strength values. This supports the given reasons why our results are lower than those reported in other studies.

Boniello et al. in their study of 6-mm, 7-mm, 8-mm, and 9-mm hamstring grafts in a sample aged 14-60 years reported tensile strength values of 2,358.8 N, 3,263.5 N, 3,907.8 N, and 4,360.3 N in the tendons, respectively, and increasing tensile strength with increasing graft diameters [19]. They also reported the patellar tendon graft to be more suitable than the hamstring tendon graft for ACL reconstruction due to its greater stability and lower failure rate.

Siegel et al. reported patellar tendon and hamstring tendon grafts (semitendinosus and gracilis tendons) to be the most commonly preferred tendons in ACL reconstruction, associating the quadriceps tendon grafts used in ACL reconstruction with significantly less anterior knee pain graft site morbidity when compared to patellar tendon grafts [2]. Görmeli et al. reported a mean thickness of 8 mm for the quadriceps tendon, which is closer to the size of the ACL, and stated that the two had similar biomechanical properties [20]. Lee et al. study of the use of quadriceps tendon autografts in 67 ACL reconstructions reported satisfactory outcomes with a reduction in donor site morbidity [21]. Garofalo et al. studied 31 patients who underwent ACL reconstruction with quadriceps tendon grafts who were followed up for at least three years after the procedure; they reported that 93% returned to sports and that none required additional surgical interventions [22]. Our study, in turn, revealed that the tendon with the lowest mean difference from the ACL in terms of tensile strength was the quadriceps tendon in both sexes, which supports the use of quadriceps tendon grafts in ACL grafts.

Limitations of the study

The limitations of this study are the limited number of grafts used and the preservation of these grafts in 10% formaldehyde solution, which may affect the structural and mechanical properties of the grafts.

## Conclusions

In conclusion, this is the first study to compare the tensile strength of ACL reconstruction and grafts used in ACL reconstruction. The data obtained in this study regarding the biomechanical properties of the ACL and the grafts used in ACL reconstruction showed that the lowest mean difference in tensile strength was found between the ACL and the quadriceps tendon. Therefore, we opine that the use of quadriceps tendon in ACL reconstruction procedures will give more favorable results. This supports the necessity of evaluating both the mechanical and structural properties of the grafts in determining the most appropriate graft to be used in ACL reconstruction.
